# Transgender Men's and Non‐Binary People's Experiences of Cervical Cancer Screening—A Journey Mapping Approach

**DOI:** 10.1111/jocn.70113

**Published:** 2025-09-19

**Authors:** Max Kleijberg, Lars E. Eriksson

**Affiliations:** ^1^ Regional Cancer Centre Stockholm‐Gotland Stockholm Sweden; ^2^ Department of Neurobiology, Care Sciences and Society Karolinska Institutet Huddinge Sweden; ^3^ School of Innovation, Design and Engineering Mälardalen University Eskilstuna Sweden; ^4^ School of Health and Medical Sciences City St George's, University of London London UK

## Abstract

**Background:**

Research indicates various barriers to cervical cancer screening for transgender people, contributing to cancer inequities. Further research is required to better understand how these barriers affect experiences along the screening trajectory, from engaging with information, through invitation and testing, to receiving test results. Research exploring how transgender people navigate these barriers is also required.

**Aim:**

To explore the experiences of cervical cancer screening in Sweden among transgender people who were assigned female at birth, and to identify touchpoints in need of improvement along the cervical cancer screening trajectory.

**Design:**

Qualitative interview study inspired by journey mapping.

**Methods:**

Semi‐structured interviews (*n* = 18) and interpretive description analysis.

**Results:**

Five phases were identified comprising participants' cervical cancer screening journey, with touchpoints in each phase indicating key experiences, barriers, and strategies to navigate barriers. Experiences of touchpoints were affected by four interrelated dimensions: The embodied person—personal gender identity, relationship with own body, and transition process; System factors—policies, routines, and practices; Gender norms and transphobia; and Prior healthcare experiences. Significant barriers included a lack of trans‐specific screening information; an invitation system that does not automatically invite male‐registered individuals with a cervix; lack of trans competency among clinics and staff; female‐centred clinics; gender dysphoria; anticipation or fear of being mistreated; distrust of healthcare authorities; and participant‐staff power dynamics.

**Conclusion:**

To make cervical cancer screening more equitable for transgender people, barriers need to be addressed by considering the four dimensions that affect these barriers.

**Implications for the Profession and/or Patient Care:**

Findings show that staff involved in policy and clinical practice can improve transgender people's experiences of cervical cancer screening by promoting agency and self‐determination in each screening phase. This involves providing inclusive information, continuing invitations for male‐registered individuals with a cervix, enhancing trans‐competency, and addressing power dynamics in staff‐participant interactions.

**Reporting Method:**

The Standards of Reporting Qualitative Research (SRQR).

**Patient or Public Contribution:**

Representatives from the Regional Cancer Centre Stockholm–Gotland were involved in the conceptualisation of this study. Representatives from trans and LGBTQI+ organisations, Regional Cancer Centres, and the National Board of Health and Welfare have provided feedback during the analysis and writing phases.

**Summary**

- Impact
  ○ In this study, we explore experiences of cervical cancer screening among transgender people who were assigned female at birth, inspired by journey mapping, to acquire knowledge that can be applied to improve cervical cancer screening for this group.
  ○ The findings describe five phases in the cervical cancer screening journey and indicate how experiences and barriers in each phase are affected by four interrelated dimensions.
  ○ The findings are clinically relevant for staff working with cervical cancer screening to facilitate improvements in the screening experience for transgender people. While the research is based in Sweden, we believe that the findings are applicable in other settings due to similar challenges in how cervical cancer screening is organised and the needs of transgender people.
- What does this paper contribute to the wider global clinical community?
  ○ The journey mapping approach introduces a novel way of understanding transgender people's experiences of and barriers to cervical cancer screening.
  ○ Findings from this study can influence policy and clinical practice to facilitate cervical cancer screening for transgender people.

## 1 Introduction

Transgender (trans) is an umbrella term for people with a gender identity and/or gender expression that does not match the sex they were assigned at birth (Brown et al. 2023; Bränström 2019). The trans population is diverse in terms of the ways individuals experience, express, and describe their gender identity (terms include e.g., trans man, trans woman, non‐binary). Transition processes, which refer to steps individuals take to live in accordance with their gender identity, also vary depending on individual needs and goals. These processes can include, for example, change of name, pronouns, and legal gender, as well as gender‐affirming care, such as hormone therapy and surgeries. Research has found that this group is affected by cancer inequities, e.g., higher cancer prevalence, later stage at diagnosis, and worse survival rates for many cancers compared to general population figures (Jackson et al. 2021; Leone et al. 2023). Studies indicate that such inequities are compounded by the barriers that trans people face regarding participation in cancer screening programmes (Grimstad et al. 2020; Leone et al. 2023; Tabaac et al. 2018). This article focuses on cervical cancer screening (CCS). Cervical cancer is often caused by certain strains of the human papillomavirus (HPV), a common virus transmitted through sexual contact, which can cause cell changes that may lead to cancer (Socialstyrelsen 2022). CCS programmes have significantly reduced cervical cancer mortality through detecting HPV and associated cell changes early (Jansen et al. 2020).

## 2 Background

The limited existing research investigating cervical cancer prevention among trans people suggests significantly lower odds of this group participating in CCS compared to the general population (Chan et al. 2024; Tabaac et al. 2018). Studies have identified various barriers that may contribute to this disparity (Connolly et al. 2020; Dhillon et al. 2020; Rivers et al. 2024). For example, while health authorities recommend CCS for anyone with a cervix (Peitzmeier et al. 2020), screening programmes often only invite people who are registered as female in their legal, medical, or insurance documents (Berner et al. 2021). This practice creates systemic barriers for male‐registered trans people with a cervix (Berner et al. 2021; Regionalt Cancercentrum Stockholm Gotland 2024; Rivers et al. 2024; Weyers et al. 2021). Consequently, there is a misalignment between CCS guidelines and CCS practices, highlighting a need to understand how CCS practice can be adjusted.

Another significant barrier is the experience or anticipation of emotional and psychological distress related to CCS as a result of gender dysphoria (Carroll et al. 2023; Dhillon et al. 2020; Rivers et al. 2024). Gender dysphoria is described as profound distress or discomfort caused by the discrepancy between assigned sex at birth and gender identity (Rivers et al. 2024). Gender dysphoria can be triggered by CCS because the screening can clash with a person's gender identity. For example, CCS is generally considered a women's examination and often takes place in female‐centred clinical environments, which can cause distress or discomfort for trans people (Berner et al. 2021; Carroll et al. 2023; Rivers et al. 2024). Furthermore, the handling of the genitals during CCS can also trigger gender dysphoria (Berner et al. 2021; Carroll et al. 2023; Rivers et al. 2024). More research is needed to better understand how gender dysphoria inadvertently triggered by CCS can be mediated.

While gender dysphoria is described as a primary reason for avoiding or delaying CCS among trans people (Dhillon et al. 2020; Rivers et al. 2024), several other experiences related to the body can also contribute to discomfort and distress during CCS. For instance, sexual trauma, which is found to be more common among trans people, can contribute to emotional and psychological distress related to CCS (Gibson et al. 2022). Additionally, some trans people are affected by previous experiences of non‐consensual medical procedures as part of their transition, which can contribute to anticipating or experiencing distress in CCS (Carroll et al. 2023). Physical pain during CCS has also been reported as a barrier, particularly among individuals receiving hormone therapy, which can cause adverse effects, such as cytological changes and vaginal mucosal atrophy that may complicate the CCS‐sampling procedure (Carroll et al. 2023; Compton et al. 2022). Experiences of negative, disrespectful, or inappropriate treatment by healthcare staff seem to be common among trans people and can deter individuals from participating in CCS (Dhillon et al. 2020; Gibson et al. 2022; Rivers et al. 2024). In some studies, trans people report experiences of a lack of trans‐related competency (i.e., knowledge and skills leading to trans‐inclusive attitudes, behaviours, practices, and policies) among healthcare staff (Berner et al. 2021; Rivers et al. 2024). Thus, research indicates barriers related to interacting with healthcare staff, bodily experiences, and CCS practices. However, more research is needed to better understand how these barriers potentially interreact and affect the diverse trans population in different ways.

While existing studies have identified various barriers, they seem to have primarily focused on experiences of the clinical aspects of CCS, such as staff‐participant interactions, experiences of female‐centric clinics, and the impact of gender dysphoria during screening. However, to improve cervical screening for trans people, knowledge is needed about the experiences and barriers along the whole CCS trajectory, i.e., from engaging with CCS information, through invitation to screening, testing (clinically and HPV self‐tests), and receiving test results. Research is also needed to explore how trans people navigate barriers along the CCS trajectory. This knowledge would enable the development of appropriate solutions along the CCS trajectory.

## 3 The Study

### 3.1 Research Aim

This study aims to explore the experiences of CCS in Sweden among trans people who were assigned female at birth, and to identify touchpoints in need of improvement along the CCS trajectory. Findings are intended to be applied to improve CCS in Sweden and are expected to be of relevance internationally.

### 3.2 Swedish Context

In Sweden, CCS was implemented in 1967, leading to an estimated 50% reduction in cervical cancer incidence (Vaccarella et al. 2014). CCS in Sweden is generally performed by midwives or gynaecologists in a clinical setting through sampling cells from the cervix. The samples are then tested for oncogenic HPV types and, when required, with cytology to detect potential cell changes. However, HPV self‐sampling through a vaginal swab has become increasingly common in recent years. If HPV is detected in the self‐sample, the person will be invited to a clinic for additional tests. CCS is offered between the ages of 23 and 70 years, with varying intervals, usually 6–7 years or shorter, depending on age and prior test results (Socialstyrelsen 2022). Although the responsibility to provide CCS in Sweden lies with the 21 autonomous healthcare regions, the formulation of CCS invitations is nationally coordinated, with efforts made to use language that is inclusive of anyone with a cervix, regardless of gender identity. Individuals are generally notified of their test results by letter.

According to directives from the National Board of Health and Welfare, CCS must be offered to all individuals “who were assigned female at birth” (Socialstyrelsen 2019). However, the current CCS invitation system is based on the female legal gender marker and does not automatically invite individuals with a cervix who were assigned female at birth but whose legal gender is currently male. In Sweden, a person's legal gender is indicated by the second‐to‐last digit in their personal identification number (personnummer, assigned by Sweden's tax agency at birth or when becoming a resident), with even numbers signifying female and odd numbers male. The personal identification number is used widely in Swedish society, including in health care. Changing legal gender, therefore, involves changing one's personal identification number. Prior to July 2025, individuals who wished to change their legal gender needed a gender dysphoria diagnosis, which required an examination by a specialised healthcare team. Under a new law effective from July 2025, this requirement has been removed. Instead, applicants must submit a certificate from a licensed physician, psychologist, psychotherapist, or healthcare counsellor.

The main official source for information about health and healthcare in Sweden is “1177”, which can be accessed by phone or digitally. In the information about HPV testing on the 1177 webpage, the system barrier for people with male legal gender and a cervix is acknowledged with the recommendation to contact the physician who was responsible for the gender dysphoria examination to request a referral for CCS.

In Sweden, Regional Cancer Centres support the country's healthcare regions in increasing cancer care quality and equity. The Regional Cancer Centre Stockholm–Gotland (RCC) has drawn attention to the system barrier to CCS for people with a cervix and male legal gender (Regionalt Cancercentrum Stockholm Gotland 2024). The research presented in this article was initiated by the RCC as part of efforts to overcome this system barrier and improve CCS experiences for trans people who were assigned female at birth.

## 4 Methods

### 4.1 Design

The research aim was addressed through an inductive qualitative interview study inspired by “journey mapping” (Davies et al. 2023; Stickdorn and Schneider 2012). Journey mapping is a method often employed in the field of service design to create or improve a service from the perspectives of those who use the service (Howard 2014; Stickdorn and Schneider 2012). It involves generating data to create a structured representation of users' interactions with a service over time (Howard 2014; Stickdorn and Schneider 2012). Various data generation methods have been used in journey mapping studies, e.g., qualitative interviews, participant observations, surveys, or a mix of methods (Davies et al. 2023). Through analysis of this generated data, touchpoints are identified (Dewar et al. 2010; Stickdorn and Schneider 2012). Touchpoints are key moments along the various phases of the service that shape users' experiences, barriers, and facilitators (Davies et al. 2023; Dewar et al. 2010; Stickdorn and Schneider 2012). Journey maps are often represented visually as a sequence of phases and touchpoints, illustrating the users' experiences along the service trajectory (Stickdorn and Schneider 2012). Journey mapping is increasingly applied in nursing and other healthcare science research to investigate and improve patient experiences of healthcare services (Davies et al. 2023; Joseph et al. 2020; Ly et al. 2021). In this study, qualitative interviews were conducted with trans people who were assigned female at birth about their experience with CCS. This data was analysed through an inductive qualitative process inspired by interpretive description (Thorne 2016) to identify touchpoints and create a journey map.

### 4.2 Recruitment

Inclusion criteria for participants were having been assigned female legal gender at birth, being at least 23 years old (the age from which CCS is recommended), and identifying as transmen or non‐binary. Recruitment began with a presentation at Stockholm Pride 2023 and continued with an advertisement through a trans organisation. Most participants, however, were recruited by word of mouth and snowball sampling (Heckathorn 2011) by people in trans communities, and through research participants sharing the invitation in trans‐specific social media groups that were otherwise inaccessible to the researchers. Potential participants were asked to contact first author MK if they were interested in participating. MK then provided them with verbal and written information about the research project, including what the interview would entail, that participation was voluntary, and the possibility of receiving financial reimbursement for participation based on an hourly rate. In total, 33 individuals contacted MK, of whom 18 participated. All were given the opportunity to ask questions prior to the interview before providing written informed consent.

### 4.3 Data Collection

Participants could choose the time and place of the interview. Three interviews were performed in‐person, at MK's office at the RCC or at a trans‐community space, and 15 were conducted virtually or via phone. The interviews were conversational in form with support from an interview guide comprising topics related to the CCS “journey” (Davies et al. 2023; Stickdorn and Schneider 2012); their length ranged from 40 to 102 min (median 69). The interview guide topics were: information related to CCS; first contact (invitation or self‐initiated); testing by CCS staff (including waiting room experience, treatment by CCS staff, ways in which gender dysphoria affected the experience of the test) or through a self‐test; test results; and expectations, fears, hopes, and needs for future CCS. Each interview began with background questions regarding demographic data, gender identity, assigned gender at birth, current legal gender, whether the participant currently has a cervix, and why the person wanted to participate in the study. This latter question generally led participants to start describing past CCS‐related experiences, after which MK used the interview guide topics to further guide the conversation. Interviews were recorded and professionally transcribed verbatim.

### 4.4 Data Analysis

The analysis was inductive based on interpretive description (Thorne 2016). Interpretive description focuses on generating knowledge that is applicable in clinical practice and encourages a pragmatic incorporation of elements from various methods (Thorne 2016), thereby supporting our research aim and use of journey mapping in the analysis. In interpretive description, data is initially coded with broad codes that are further developed and fine‐tuned through an iterative coding process to identify patterns and themes in the data (Thorne 2016). MK led the analysis in a process with regular reflexive and analytic meetings with LEE, and with support from a research assistant in the initial stage. MK kept reflexive and analytic notes throughout this process (Thorne 2016).

MK listened to all interviews while reading the transcripts to make corrections, gain a deeper understanding of the dataset, and make analytic notes about initial ideas for describing various phases of the CCS journey. These notes were discussed with LEE and the research assistant. MK and the research assistant then together discussed and generated initial broad codes from analysing a first interview. After this, MK continued coding all interviews in an iterative and inductive process. In line with interpretive description, this process entailed going back and forth between different parts of the data to explore various interpretations of service phases and touchpoints (Thorne 2016). Through this process, the codes were further developed and fine‐tuned to eventually describe CCS phases as experienced and identify touchpoints in each phase and dimensions affecting the experience of these touchpoints. Preliminary findings were discussed with RCC representatives, national and international cancer and health equity researchers, and representatives from the National Board of Health and Welfare, as well as with trans and LGBTQI+ organisations. These discussions facilitated the final conceptualisation of the findings.

### 4.5 Rigour and Reflexivity

Efforts were made to recruit participants with diverse demographic backgrounds and gender identities as these characteristics might affect the experience of CCS. The study was led by first author MK, a researcher with a background in design and healthcare science research, with support from last author LEE, a professor in nursing. Both have expertise in research regarding sexual and gender minority groups and collaborated closely in this project. The research process was reflected upon in regular meetings among researchers as well as with RCC representatives. Furthermore, as noted above, preliminary findings were discussed with various stakeholders throughout the analysis to critically reflect on the coding process and formulation of findings. This study adheres to the Standards for Reporting Qualitative Research (SRQR) checklist (Data S1).

### 4.6 Ethical Considerations

Ethical approval for this study was obtained from the Swedish Ethical Review Authority (2022‐03292‐01, 2023‐04330‐02).

## 5 Findings

### 5.1 Characteristics of Participants

The ages of the 18 participants ranged from 24 to 60 (Median = 36) years. Nine had male legal gender, and some remaining participants were in the process of changing to male legal gender. Thirteen participants described themselves as transmen or men, five as non‐binary (also using terms such as transmasculine and agender). Two participants had undergone a total hysterectomy after the age of 23, as part of their gender‐affirming care. They had experience of participating in or being invited to CCS prior to their hysterectomy; thus, interviews focused on these experiences. Participants lived in eight of the 21 Swedish healthcare regions, most in or around the three largest cities, while others lived in smaller towns or in remote areas. Fifteen participants were born in Sweden. Fourteen had some form of higher education; four had completed high school. Seven participants had health or care‐related professions, while others had various other professions or were students.

### 5.2 The Cervical Cancer Screening Journey Map

**FIGURE 1:**
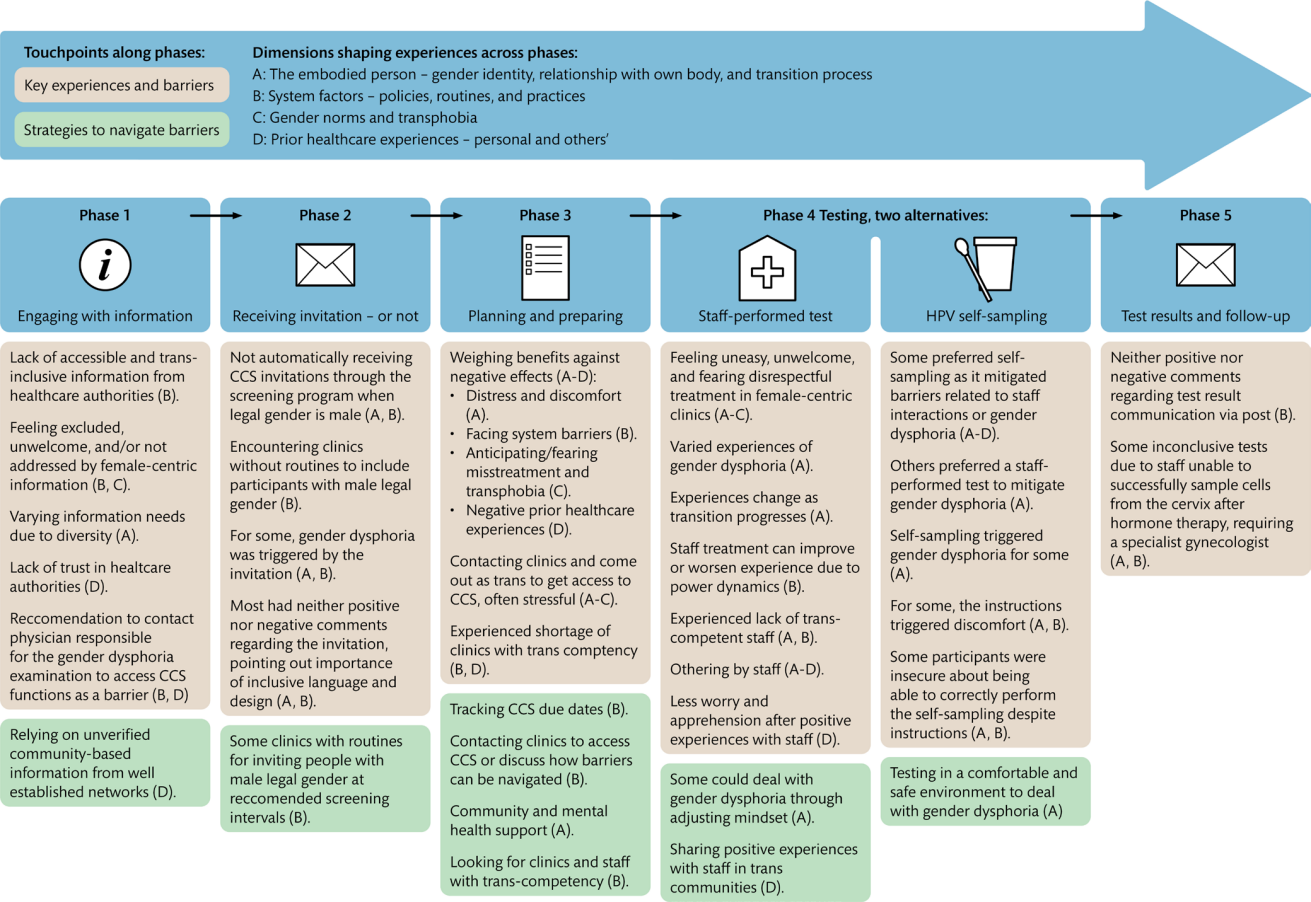
CCS journey map based on analysis of interviews with trans people in Sweden who were assigned female at birth. [Colour figure can be viewed at wileyonlinelibrary.com]

Figure 1 illustrates the journey map based on the data analysis. Five phases were identified: Phase 1, Engaging with CCS‐related information; Phase 2, Receiving an invitation to CCS—or not; Phase 3, Planning and preparing for CCS participation; Phase 4, Testing alternative 1, participating in a staff‐performed test; Phase 4, Testing alternative 2, HPV self‐sampling; Phase 5, Receiving test results and potential follow‐up (Figure 1). The figure illustrates touchpoints for each phase, i.e., participants' key experiences and barriers, and strategies to navigate barriers. Four dimensions (A‐D) were found that shape experiences along participants' CCS journey (Figure 1). Some touchpoints were described by participants as affected by more than one dimension (Figure 1), indicating the interrelation of dimensions. We describe the dimensions first separately for clarity, after which we present the five phases with related touchpoints and how the dimensions influence them.

### 5.3 Interrelated Dimensions Shaping Experiences Along the Cervical Cancer Screening Journey

#### 5.3.1 A. The Embodied Person—Gender Identity, Relationship With Own Body, and Transition Process

Participants described their experiences of CCS in relation to their gender identity and expression, relationship with their body (including experiences of gender dysphoria), and transition process. We refer to this dimension as *the embodied person* to reflect participants' accounts of how their CCS experiences were affected by how they feel in and about their bodies. The diversity among participants regarding these areas led to a wide variety of CCS experiences and barriers described. For example, participants who described pronounced gender dysphoria in relation to their genitals experienced different barriers from participants who described experiencing a lesser degree of gender dysphoria. The nature of transition processes also varied among participants (e.g., name change, legal gender change, hormone therapy, surgeries), leading to different barriers described by participants, for example between those with male legal gender and those with female legal gender. Participants further noted that their CCS experiences changed as their transition progressed, describing different experiences and barriers with increasing masculine presentation.

#### 5.3.2 B. System Factors—Policies, Routines, and Practices

All participants described how system factors such as policies, routines, and practices could hinder or facilitate their CCS journey. Such system factors exist at national and more local levels. A barrier at national level described by participants is that individuals with a cervix and male legal gender do not automatically receive invitations through the CCS programme. On more local levels, participants noted that clinics manage this issue inconsistently or not at all. System factors described by participants also include policies, routines, and practices regarding CCS information, testing, and appointment‐booking systems. Participants explained that system factors affected their CCS experiences differently depending on their gender expression and transition process (e.g., not automatically receiving CCS invitations after changing legal gender), illustrating an interrelationship with dimension A, the embodied person.

#### 5.3.3 C. Gender Norms and Transphobia

All participants appeared to reflect on how societal gender norms affected their CCS experience, with participants particularly referring to the prevailing assumptions that gender is binary (female or male) and aligns with biological sex (or sex assigned at birth). The impact of these norms on participants' CCS experiences is evident along the CCS journey, with participants describing that information from healthcare authorities does not include trans perspectives, that they have to come out as trans to access CCS, and that testing occurs in female‐centric clinics. This dimension is closely related to system factors (B), since gender norms were described as influencing CCS policies, routines, and practices. This dimension also encompasses the impact of participants describing experiencing transphobia, including prejudice and disrespectful treatment based on trans identity or non‐conformity to gender norms, illustrating an interrelationship with dimension A, the embodied person.

#### 5.3.4 D. Prior Healthcare Experiences—Personal and Others'

All participants described how their own or other trans people's prior healthcare experiences, including gender‐affirming care, gynaecological care, and healthcare in general, affected their CCS expectations and experiences. Many participants described having low trust in healthcare services due to prior negative experiences, while prior positive experiences seemed to mitigate barriers related to participants' expectations of transphobia or staff's lack of trans competency. Some participants noted that negative prior healthcare experiences affected their transition process and their relationship with their body, illustrating an interrelation with dimension A, the embodied person. System factors (B) such as policies and practices were also frequently described as affecting prior healthcare experiences. Furthermore, participants often substantiated experiences of gender norms and transphobia (C) with examples of disrespectful treatment in prior healthcare experiences.

### 5.4 Touchpoints Along the Cervical Cancer Screening Journey

Below we describe findings in relation to each CCS phase. We refer to the touchpoints illustrated in Figure 1 (i.e., key experiences and barriers, and strategies to navigate barriers) as well as how these are affected by dimensions A–D (Figure 1).

#### 5.4.1 Phase 1. Engaging With CCS‐Related Information

Participants generally noted a lack of trans‐inclusive CCS information from healthcare authorities, pointing to barriers affected by system factors (B, Figure 1) regarding CCS information provision. CCS information provision also seemed influenced by gender norms (C, Figure 1), as some participants explained that CCS information is often female‐centric, which made them feel excluded, unwelcome, and/or not addressed. Needs for information varied among participants due to varying transition processes (A, Figure 1), with male‐registered persons particularly expressing a need for information about how to access CCS. More generally, participants talked about needing information about clinics and staff with trans competency.

As a strategy to deal with a lack of information from healthcare authorities, many participants described relying on information from well‐established trans‐communities, often through social media. Another reason for relying on community‐based information was said to be a lack of trust in the healthcare system based on prior negative experiences (D, Figure 1). Whereas participants generally talked about community‐based information as being reliable, since it is shared by people with similar experiences and through already existing networks, such information was also critically evaluated rather than accepted at face value, as illustrated in this quote:

Trans man in his 30's: It could be a hoax, because there's a lot of that in these circles, but I know that someone said that trans guys should get tested more often than others because we're at a higher risk, that we should [get tested] every year. But that's nothing I've found confirmed anywhere in any article.

Even when directly asked, none of the participants described having seen information on the 1177 website (see Swedish context section) recommending contact with the physician responsible for the gender dysphoria examination to access CCS. Participants noted that the 1177 information is not easily accessible and does not show up in internet searches using terms such as trans or non‐binary. Generally, participants pointed out that this recommended procedure functions as a barrier because it adds an extra step to accessing CCS. Some described experiencing trans care as being hard to reach and overburdened, whereas others had only interacted with trans care a long time ago or said they would rather avoid future contact due to prior negative experiences. Thus, participants seemed to experience this recommended procedure as a barrier affected by both system factors and prior healthcare experiences (B and D, Figure 1).

#### 5.4.2 Phase 2. Receiving an Invitation to CCS—or Not

Participants described receiving or not receiving an invitation to staff‐performed CCS and/or an HPV self‐test as an important touchpoint. Participants talked about the CCS programme only automatically inviting people with female legal gender as a system barrier for people with male legal gender (A and B, Figure 1). One participant demonstrated the importance of such practical issues, saying:

Trans man in his 30's: That's one of the reasons for me not to change my legal gender, because I don't have the energy to call the healthcare centre regularly […] It's much easier to just wait until they send an invitation.

Most male‐registered participants had experiences of clinics without routines to address this issue (B, Figure 1), as exemplified by a participant with experience of CCS in different towns:

Trans man in his 60's: A few months ago, in [city X], I met a midwife who informed me very well and clearly that I will not receive [a CCS invitation], but that I need to contact [the clinic] myself in a few years. And in [city Y], it didn't happen automatically either, but then they said that I could be on a list so that I would get invitations, but I didn't receive invitations anyway. So, after a few years I contacted [them] myself.

However, two participants with male legal gender described that their clinics seemed to have strategies to address this barrier as these clinics each routinely invited them at the recommended screening intervals. Both explained that it was unclear to them how this routine was established and were unsure if this would change clinics. Thus, this strategy appears to be informed by local system factors at these clinics (B, Figure 1).

Some described that getting a CCS invitation could trigger their gender dysphoria, which could be a reason for them to avoid or postpone CCS participation. However, most participants who received CCS invitations reported reacting neither negatively nor positively to the way the invitation was designed or formulated, with some pointing out the importance of the invitation being inclusive to trans people with a cervix, so that people in this group understand that they are included in CCS. Thus, the experience of this touchpoint seems to be affected by both the embodied person dimension (e.g., potentially triggering gender dysphoria) and system factors informing the formulation and design of CCS invitations (A and B, Figure 1).

#### 5.4.3 Phase 3. Planning and Preparing for CCS Participation

To decide whether to participate in CCS or not, participants described weighing the potential benefits (i.e., cervical cancer prevention and early detection) against potential negative effects in relation to the four dimensions (A–D, Figure 1). For example, some participants said they avoided or postponed CCS due to distress and discomfort in relation to their own body and/or gender identity (A, Figure 1). Particularly, participants with male legal gender talked about needing to navigate system barriers to access CCS (B, Figure 1). Some talked about fearing or anticipating mistreatment and transphobia by CCS staff (C, Figure 1). Some participants also explained that negative prior healthcare experiences, such as with gender‐affirming care, led them to deprioritise CCS (D, Figure 1). Often, participants considered a combination of dimensions as illustrated below.

Those who decided to participate described a need for planning and preparation on their part to mitigate potential negative effects and navigate barriers. For participants with male legal gender, planning and preparation efforts entailed actively keeping track of when they were due for CCS and contacting clinics to get access to screening to navigate system factors (B, Figure 1). Participants explained that this requires them to come out as trans, which was generally described as stressful and draining in a societal context where they fall outside prevailing gender norms and may encounter transphobia, illustrating a barrier affected by three dimensions, i.e., the embodied person, system factors, and gender norms (A–C, Figure 1). Preparing for navigating this barrier could include mental preparation, community support, and professional mental health support (Figure 1).

One participant with male legal gender who described having had traumatising healthcare experiences in the past, talked about not having been able to participate in CCS as staff at the facility he contacted required a certificate from a healthcare authority confirming his CCS eligibility. Despite him actively trying to fulfil this demand, he was unable to obtain such a certificate. He described his experience with the clinic and healthcare authority as transphobic as they seemed unwilling to support him, which discouraged him from further efforts to participate in CCS. This is an example of how barriers were created for this participant through interrelated dimensions, as system factors regarding access to CCS (B) were influenced by gender norms (C), excluding this participant who falls outside such norms (A), compounded by his prior traumatic healthcare experiences (D). It should be noted that other participants with male legal gender, who had contacted clinics to participate in CCS, described being met professionally and were able to schedule CCS participation, illustrating the wide variety of experiences among participants and ways in which clinics dealt with the system barrier.

Many participants described looking for clinics and staff with trans inclusive practices and routines to mitigate the anticipation or fear of transphobia (B, Figure 1). Strategies included enquiring in trans communities, requesting recommendations from other healthcare instances, and searching for “LGBTQI‐certified” clinics (i.e., clinics that have invested in education to increase sexual and gender minority competency among staff). Many participants noted a shortage of clinics and staff with trans competency in their area. Some explained that they would call clinics in advance, both to understand whether the clinic has trans competency, but also to prevent uncomfortable situations (B, Figure 1):

Trans man in his 20's: It's kind of a defence, I don't want to end up in a difficult situation, so I prepare by [calling] first. […] Because they maybe see a name in my healthcare records, but they'll see that [my legal gender] is female. So, then they won't really know what to write. […] And it's a bit of a strategy, I don't want to create confusion that can lead to an uncomfortable situation for myself as a trans person.

Other concerns that participants mentioned preparing for were fearing/anticipating transphobia and/or being outed as trans in waiting room situations. Besides prior healthcare experiences (D) and societal gender norms (C), participants related such fear to routines and practices in clinics (B), e.g., CCS generally taking place in female‐centred clinical settings and encountering other CCS participants in waiting room settings. Two participants described having addressed these concerns with staff, leading to solutions such as after‐hours appointments for privacy or screening at an all‐gender sexual health clinic. Some participants from smaller towns were worried about being recognised. They said they planned to travel to clinics further away to assure anonymity. Others discussed bringing along a companion for support to reduce the fear of transphobic encounters.

#### 5.4.4 Phase 4. Alternative 1: Participating in a Staff‐Performed Test

Besides experiences of the staff‐performed test, participants also discussed related touchpoints, such as their impressions of the clinic and waiting room experiences. Several participants described that, when the clinic presented itself as a “women's clinic”, they felt uneasy, unwelcome, and/or feared disrespectful treatment by staff or others in the space:

Non‐binary person in their 30's: It's mostly worry, this feeling that you're sort of intruding in a women's space. It's supposed to be safe for women to go there and check their genitals and stuff, and it feels like you're not supposed to be there because you're masculine‐coded. […] I don't want to be subjected to transphobia by others in the [waiting room], for example the women, because they have prejudices about trans people, and they might think a man with a vagina is disgusting […] But it could also be that I stress myself out unnecessarily. That I take… this transphobia that's around, that I sort of absorb it and kind of adopt it in a space where it doesn't have to be that way, because… I mean, they're just there to get their genitals checked.

This quote illustrates how experiences of a female‐centric waiting room are affected by the interrelation of several dimensions, as this participants explained navigating feelings of worry, uncertainty, and intrusion based on presenting masculine (A, the embodied person) in a female‐centric CCS space (B, clinical routines and practices), along with understanding that they are allowed to be in this space and that their fears are amplified by gender norms and transphobia in society (C, Figure 1). While several participants described a fear of transphobia in the waiting room, no one described having encountered this. Generally, participants spoke of wishing that clinics would present themselves as trans‐inclusive through symbols such as the trans‐flag, as this could help make them feel more welcome and at ease. A few participants described their participation in CCS as “quiet activism”, since their presence in traditionally female‐centric spaces could serve as a reminder of the existence of trans people.

Experiences of the staff‐performed test were partly affected by the various ways participants related to their own body and experienced gender dysphoria (A, Figure 1). For some, gender dysphoria triggered by testing was the main reason for not participating, while for others, gender dysphoria was manageable or entirely unproblematic during testing. Several participants described that they dealt with testing‐related gender dysphoria through a mindset that can be characterised by resilience, determination, and a pragmatic acceptance of the temporary distress/discomfort, exemplified by one participant saying: “You end up in a mindset that this has nothing to do with me, that now I just have to do this thing and I can think about myself another time” (A, Figure 1).

Participants also reflected on how their transition process and its progress affected their experiences in the clinical setting (A, Figure 1). Participants who described themselves as generally being perceived as women reported that this perception facilitated their CCS experiences as they did not have to anticipate transphobia. Conversely, those who described themselves as appearing more masculine often related this to feeling uneasy or out of place in the eyes of others and anticipating or fearing transphobia. However, a few participants mentioned that their CCS experience improved as their appearance became more masculine, exemplified by the following quote:

Trans man in his 30's: The first time it was incredibly uncomfortable that I was seen as a girl. But [this time] the midwife was very surprised, and I saw that she was like “what should I say now?” She was very unsure. Then she was very professional and did it well […] She said, “so you have internal genital organs that are female?” “Exactly” I said, and I felt so affirmed by that […] you see, she didn't say “So you're born a woman?” or “You're actually a woman?” […] It affirmed me so much, so it wasn't so hard.

Many participants discussed that the way they felt treated by CCS staff was an important factor that could either improve or worsen their testing experience, as exemplified by the following quote:

Non‐binary person in their 20's: I think both times they were LGBTQI‐certified, but only the second time […] was much better because then it felt like the person at least informed me […] and checked “do you really want to do this? It's up to you, it doesn't matter if it's important or not, if you don't want to do it then you shouldn't do it”. So, it felt like I had a choice […] It felt like I had something to say about it […] It feels better if you have the possibility to say no and stop the process. Because if no one tells me that I can say no, I don't know that it's an option.

The quote illustrates power dynamics between staff and the CCS‐participant based on staff routines and practices (B, Figure 1). This participant described gaining agency and control through the communication with staff. In contrast, a few participants felt coerced into CCS during gynaecological appointments designated to discussing gender‐affirming care. One of these participants said that the unplanned test felt like a violation, raising issues of consent. A few participants discussed how their neurodiversity intersected with their trans‐experiences and highlighted the importance of clear and pedagogical communication from staff to build trust and increase their agency.

Many participants described a lack of competency among CCS staff and other healthcare professionals regarding gender dysphoria, gender identity, the effects of hormone therapy and surgeries, and how these can affect CCS, showing an interrelation between the embodied person dimension and staff routines and practices (A and B, Figure 1). Several participants mentioned, for example, that their hormone therapy had led to fragile mucous membranes, complicating CCS and making it more painful. Some explained that this barrier would be easier to navigate if staff were more knowledgeable. In the quote above, the participant mentions that although both clinics they visited were LGBTQI‐certified, they only had a positive experience at one, indicating that such certifications do not guarantee trans competency or respectful treatment, an issue also raised by other participants.

Several participants described interactions with staff who asked about their trans identity and transition process, questions unrelated to CCS or the care appointment. Most participants described feeling uncomfortable with such questions, noting that this contributed to their sense of being othered and acted as a barrier to CCS participation related to all four dimensions, i.e., the embodied person, staff practices, gender norms, and prior healthcare experiences (A–D, Figure 1). However, others welcomed the opportunity to educate staff who showed a genuine willingness to learn. Generally, participants described that good treatment by CCS staff meant being treated like any other individual participating in screening.

While some participants spoke of negative experiences with CCS staff, several noted only positive interactions. Others, initially apprehensive about getting tested due to fear of mistreatment, described becoming progressively less worried after having positive experiences with staff (D, Figure 1). One participant noted the importance of sharing such positive healthcare experiences through trans communities, “so that people don't go around being afraid unnecessarily” (D, Figure 1).

#### 5.4.5 Phase 4. Alternative 2: HPV Self‐Sampling

Some participants had experience of self‐sampling, which most preferred. Those without experience of self‐sampling often said they would prefer it. A self‐sampling preference was explained by some as it mitigates barriers related to fearing or anticipating transphobia or mistreatment in staff interactions sometimes based on personal or others' prior healthcare experiences. This preference is thus affected by all four dimensions, i.e., the embodied person interrelated with staff practices, gender norms and transphobia, and prior healthcare experiences (A–D, Figure 1). Others noted that self‐sampling allowed them to better deal with their gender dysphoria, e.g., by being in a comfortable space rather than in a clinical setting (A, Figure 1). However, a few participants talked about having postponed their self‐sampling as it triggered their gender dysphoria, indicating the variety among participants based on different experiences of gender dysphoria (A, Figure 1). Some also mentioned that they preferred staff sampling as this would give them the opportunity to ask questions regarding the effects of their hormone therapy.

Some participants described feeling insecure about being able to correctly perform the self‐sampling and preferred staff‐sampling. This seemed to be related to communication issues regarding the instructions provided with self‐sampling kits. One participant described reacting negatively to the instructions, saying:

Non‐binary person in their 20's: I haven't done my self‐test yet, because I opened the envelope and my body kind of shivered. […] It says: This swab, you shouldn't stick it up all the way to the cervix or whatever it says, it should just go in 5 cm. And I was like, 5 cm is a lot! It's not a little! Just looking at the content made my body kind of cringe, because it just feels wrong.

This quote illustrates that the experience of the instructions was affected by the interrelation of routines and practices regarding the formulation of instructions and the embodied person dimension (A and B, Figure 1).

#### 5.4.6 Phase 5. Receiving Test Results and Potential Follow‐Up

Participants generally described receiving test results via post and made little comment regarding this practice (B, Figure 1). Most participants said that they received negative HPV test results. Two mentioned that cell changes were detected and that they had follow‐up testing, which they described as unproblematic. Two participants had experience of inconclusive tests performed in clinical settings, as CCS staff were unable to successfully sample cells from the cervix. In both cases, they were told that this was likely due to changes after hormone therapy. Both were offered the possibility of sampling with a specialist gynaecologist, indicating the existence of follow‐up routines after inconclusive tests related to gender‐affirming care (A and B, Figure 1).

## 6 Discussion

This study explored the experiences of CCS in Sweden among trans people who were assigned female at birth and identified touchpoints in need of improvement along the CCS journey. We have done this through a qualitative interview study inspired by journey mapping. Through analysis of interviews with 18 trans people (including trans men and non‐binary individuals), we identified five phases composing their CCS journey and four interrelated dimensions that affect CCS touchpoints: the embodied person, system factors, gender norms and transphobia, and prior healthcare experiences (Figure 1).

Other studies have distinguished barriers at individual (e.g., gender dysphoria), interpersonal (e.g., staff treatment), and system levels (e.g., programmes based on legal/registered gender) (Connolly et al. 2020; Rivers et al. 2024; Weyers et al. 2021). Rivers et al. (2024) pointed out that barriers at these various levels can amplify each other. Our findings show the ways in which this happens through the interrelation between the four dimensions. For instance, gender dysphoria‐related barriers may be seen as existing at an individual level, but in our findings, they were not only shaped by an individual's gender identity and relationship with their body (A), as they were also exacerbated by system factors, such as CCS routines and practices (B), societal gender norms and transphobia (C), and negative prior healthcare experiences (D). Similarly, barriers at interpersonal levels along the CCS journey were influenced not only by CCS staff upholding societal gender norms (C) in interactions with individuals outside these norms (A), but were also affected by CCS policies, routines, and practices (B) and participants' prior healthcare experiences (D). Based on our findings, we argue, therefore, that addressing barriers along the CCS journey requires coordinated strategies and efforts that consider the interrelation between the four dimensions.

Another contribution of this study is the identification and description of a distinct “planning and preparing” phase for participants in relation to CCS. All participants indicated the necessity of planning and preparing for CCS in order to navigate barriers. We have not found other research that highlights this, although it is likely not unique to the Swedish context, since the underlying reasons for planning and preparation efforts have been noted in other studies, i.e., not automatically receiving a CCS invitation due to registered gender, gender dysphoria triggered by CCS, fearing mistreatment by staff, and anticipating transphobia in female‐centric clinics (Connolly et al. 2020; Rivers et al. 2024; Weyers et al. 2021). We believe, therefore, that our findings have broader applicability. It is essential for healthcare providers to understand this planning and preparation phase, since our findings show that they can support these efforts, e.g., by facilitating the participation of individuals registered as male, providing trans‐specific information, and offering alternatives to female‐centric clinics.

It is striking that a significant barrier seemed to be the anticipation of negative experiences in the various CCS phases, as also pointed out by other studies (Connolly et al. 2020; Rivers et al. 2024). While anticipation of negative experiences can be perceived as an individual‐level barrier, our findings indicate that this should be systemically addressed, since this anticipation is often caused and/or amplified by societal gender norms and transphobia (C) and negative prior experiences (D). This may be explained through the minority stress model, which describes how exposure to stigma and discrimination throughout life leads individuals to anticipate future negative experiences, leading to a chronic form of stress (Meyer 2015). In our findings, this stress is expressed by e.g., participants anticipating or fearing transphobic interactions in various CCS phases. It should be noted, however, that minority stress can also lead to resilience as individuals develop coping strategies to deal with adversity (Meyer 2015). This was particularly reflected in the planning and preparation phase, in which participants described various strategies they used to avert potential negative experiences. In addition, many participants described well‐established trans community groups that offer both social support and fora in which to exchange relevant experiences and information, examples of what Meyer (2015) termed collective resilience.

Our findings also indicate participant‐staff power dynamics that can create barriers to participation. These findings corroborate research by Peitzmeier et al. (2020) who investigated power dynamics in CCS between staff and trans people assigned female at birth. Findings from both Peitzmeier et al. (2020) and our study indicate that such power dynamics are enacted by, e.g., staff questioning participants' experiences, staff not facilitating CCS for those registered as male, or staff pressuring individuals to participate against their will. Conversely, our findings and those of Peitzmeier et al. (2020) indicate that staff can also empower participants, for example by acting empathically, asking for consent repeatedly throughout CCS, and providing participants with options for adjustments. While some participants may advocate for their needs, which can address the participant‐staff power dynamic (Peitzmeier et al. 2020), it is important that CCS staff are aware of their position of power, so they do not unwittingly create barriers and are able to take actions to positively impact the overall CCS experience for trans people.

Addressing power dynamics in care settings would make CCS more equitable for trans people. Browne et al. (2018) described equity‐oriented healthcare as an approach that aims to reduce the effects of structural inequities, stigma, and mismatches between care practices and the needs of people affected by inequities. In their model of equity‐oriented care, Browne et al. (2018) describe “contextual tailoring” as a vital process through which staff tailor services to the specific needs of people affected by inequities, taking into account their often evolving context. We believe that the concept of contextual tailoring is applicable to CCS for trans people, since we found that the CCS experiences were highly individual due to personal contexts and that these experiences change over time as transition processes progress. Contextual tailoring may help staff to become aware of the diverse and evolving experiences among trans people and tailor CCS to their specific needs.

### 6.1 Strengths and Limitations of the Work

A strength of this study is its novel journey mapping approach to CCS. This facilitated a detailed and deep understanding of experiences and barriers along the CCS trajectory by making a distinction between the CCS phases and dimensions that affect CCS experiences in these phases. Another strength is the diversity of participants in terms of gender identity, experiences of gender dysphoria, transition process and progress, and demographic characteristics. However, when interpreting findings, it should be remembered that participants were often recruited through word‐of‐mouth and perhaps, as a result, include those who generally found it important to participate in CCS. Only one participant noted never having participated in CCS due to various barriers. This perspective may be underrepresented in this research, since previous studies have noted that many trans people do not participate in CCS (Chan et al. 2024; Tabaac et al. 2018). Furthermore, researcher bias could influence the interpretation of data even though efforts were made to address this, e.g., by discussing preliminary findings with a broad range of community and healthcare stakeholders.

### 6.2 Implications for Policy and Practice

In order to facilitate CCS for trans people, barriers and touchpoints in need of improvement should be addressed, taking into account the interrelation between the four dimensions (Figure 1). Clinics and staff should increase trans competency, which, based on our findings, should include knowledge about how CCS barriers and experiences can be affected by gender identity, gender dysphoria, and gender‐affirming care (dimension A); policies, routines, and practices (dimension B); gender norms and transphobia (dimension C); and prior (traumatic) healthcare experiences (dimension D). Furthermore, such competency should include an understanding of ways in which minority stress and participant‐power dynamics can affect CCS experiences. Trans competency should lead to staff having and applying tools to help mitigate barriers and facilitate the CCS experience for trans people. The findings also indicate that clinics can help trans people feel welcome and at ease by signalling that they are trans‐inclusive and have trans competency, for example through LGBTQI+ certification and the use of trans symbols. However, it is crucial that these efforts are genuinely integrated into the clinic's policies and practices and not merely performative. In addition, the findings indicate a need for healthcare authorities to provide trans‐relevant information regarding CCS and CCS participation. We suggest that such information is provided and disseminated in accessible ways and in relevant contexts in partnership with trans community groups and organisations. Finally, it is important that individuals continue to receive CCS invitations after they register as male. This implies that policies at a national level, and routines and practices in clinics, are inclusive. In summary, these efforts should aim to increase the agency and self‐determination of trans people along the CCS trajectory.

### 6.3 Recommendations for Further Research

While this study contributes to a deeper understanding of the experiences and barriers along the CCS trajectory for trans people assigned female at birth, population‐based studies are needed to better understand CCS screening behaviours among trans people. In response to the above‐mentioned implications for policy and practice, improving the CCS experience for trans people requires research to understand the support that staff and clinics need to implement trans‐inclusive CCS and to investigate the impact of CCS policy, routines, and practice interventions. We recommend that future research regarding cervical cancer prevention should include experiences and barriers related to HPV vaccination programmes, since some participants raised vaccine‐related issues even though this was not included in the interview guide, as it only focused on CCS.

## 7 Conclusion

This research contributes with a journey mapping perspective on the experiences with CCS in Sweden among trans people who were assigned female at birth that allowed identification of touchpoints in need of improvement. A significant barrier is a system that does not automatically include people with male legal gender and a cervix. Furthermore, participants reported a lack of trans‐specific, accessible, and trustworthy information from healthcare authorities. Other significant barriers discussed were a perceived lack of trans competency among clinics and staff and a fear of being mistreated. Furthermore, participants had to navigate gender dysphoria in several CCS phases and power dynamics in their interactions with healthcare staff. All participants indicated making efforts to access CCS and improve their experience of CCS, described in a distinct planning and preparation phase. Findings indicate that experiences along the CCS trajectory are affected by four interrelated dimensions: the embodied person (dimension A); system factors (dimension B); gender norms and transphobia (dimension C); and prior healthcare experiences (dimension D). Therefore, to make CCS more equitable for trans people, practice and policy changes need to take these dimensions, and how they affect each other, into account. While clinics and staff arguably play an important role in this work, more research is required to understand the support they need to this end. Furthermore, while many findings of this research are in line with other qualitative research in this field, more population‐based research is needed to better understand CCS screening behaviour among trans people and how this compares to the general population.

## Author Contributions

M.K. conceptualised the project with feedback from L.E.E. and representatives from the Regional Cancer Centre Stockholm‐Gotland. Recruitment of research participants, data generation, and data analysis were led by M.K. Throughout these phases, L.E.E. provided feedback, advice, and input through reflective discussions. M.K. led the writing process and wrote the original draft, which was iteratively edited through feedback from L.E.E. Both authors have approved the version to be published.

## Conflicts of Interest

The authors declare no conflicts of interest.

## Supporting information


**Data S1:** jocn70113‐sup‐0001‐SRQR_Checklist.pdf.

## Data Availability

The data underlying this study are not publicly available due to privacy and ethical restrictions.
